# EXpanding Treatment for Existing Neurological Disease (EXTEND): An Open-Label Phase II Clinical Trial of Hydroxyurea Treatment in Sickle Cell Anemia

**DOI:** 10.2196/resprot.5872

**Published:** 2016-09-12

**Authors:** Angela E Rankine-Mullings, Courtney R Little, Marvin E Reid, Deanne P Soares, Carolyn Taylor-Bryan, Jennifer M Knight-Madden, Susan E Stuber, Asha V Badaloo, Karen Aldred, Margaret E Wisdom-Phipps, Teresa Latham, Russell E Ware

**Affiliations:** ^1^ Sickle Cell Unit Tropical Medicine Research Institute University of the West Indies Kingston Jamaica; ^2^ Division of Hematology Cincinnati Children’s Hospital Medical Center Cincinnati, OH United States; ^3^ Tropical Metabolism Research Unit Tropical Medicine Research Institute University of the West Indies Kingston Jamaica

**Keywords:** ultrasonography, Doppler, transcranial, stroke, anemia, sickle cell, child

## Abstract

**Background:**

Cerebral vasculopathy in sickle cell anemia (SCA) begins in childhood and features intracranial arterial stenosis with high risk of ischemic stroke. Stroke risk can be reduced by transcranial doppler (TCD) screening and chronic transfusion therapy; however, this approach is impractical in many developing countries. Accumulating evidence supports the use of hydroxyurea for the prevention and treatment of cerebrovascular disease in children with SCA. Recently we reported that hydroxyurea significantly reduced the conversion from conditional TCD velocities to abnormal velocities; whether hydroxyurea can be used for children with newly diagnosed severe cerebrovascular disease in place of starting transfusion therapy remains unknown.

**Objective:**

The primary objective of the EXpanding Treatment for Existing Neurological Disease (EXTEND) trial is to investigate the effect of open label hydroxyurea on the maximum time-averaged mean velocity (TAMV) after 18 months of treatment compared to the pre-treatment value. Secondary objectives include the effects of hydroxyurea on serial TCD velocities, the incidence of neurological and non-neurological events, quality of life (QOL), body composition and metabolism, toxicity and treatment response, changes to brain magnetic resonance imaging (MRI) and magnetic resonance angiography (MRA), genetic and serologic markers of disease severity, and cognitive and pulmonary function.

**Methods:**

This prospective Phase II trial will enroll children with SCA in Jamaica, between the ages of 2 and 17 years, with either conditional (170-199 cm/sec) or abnormal (≥ 200 cm/sec) TCD velocities. Oral hydroxyurea will be administered daily and escalated to the maximum tolerated dose (MTD). Participants will be seen in the Sickle Cell Unit (SCU) in Kingston, Jamaica monthly until achieving MTD, and then every 3 months. TCD will be performed every 6 months.

**Results:**

Currently, 43 participants have been enrolled out of a projected 50. There was one withdrawal due to immigration, with no permanent screen failures. Of the 43 enrolled, 37 participants have initiated study treatment.

**Conclusions:**

This trial investigates the effects of hydroxyurea treatment at MTD in children with conditional or abnormal TCD velocities before transfusion therapy and may represent an important advance towards establishing a suitable non-transfusion protocol for stroke prevention in children with SCA. The trial outcomes will have profound significance in developing countries where the disease burden is highest.

**ClinicalTrial:**

ClinicalTrials.gov NCT02556099; https://clinicaltrials.gov/ct2/show/NCT02556099 (Archived by WebCite at http://www.webcitation.org/6k1yMAa9G)

## Introduction

Sickle cell disease (SCD) is an inherited hematological disorder that is appropriately considered a major global public health problem [1]. Although Africa bears the highest burden, SCD occurs worldwide including the United States, the Caribbean, Central and South America, the Mediterranean region, and India [2,3]. The most common and severe genotype of SCD is homozygous sickle cell anemia (HbSS), usually referred to as sickle cell anemia (SCA). Manifestations of SCA include chronic hemolytic anemia, frequent pain and other vaso-occlusive complications including acute chest syndrome, widespread organ damage, and early mortality.

Stroke is a particularly devastating complication of SCA. At the University of the West Indies (UWI), strokes occurred in 17 of 310 children with SCA followed from birth, representing an incidence of 7.8% by 14 years of age [4]. In the US Cooperative Study of Sickle Cell Disease, the cumulative incidence of primary stroke in SCA was 11% by age 20 years [5]. Even with prompt recognition and emergency medical management, the clinical sequelae of strokes are significant with frequent motor and neurocognitive deficits.

Risk factors for ischemic stroke include prior transient ischemic attack, low steady-state hemoglobin concentration, rate of and recent episode of acute chest syndrome, and elevated systolic blood pressure [5]. However, an elevated transcranial Doppler (TCD) velocity is the strongest known risk factor for stroke in SCA; in particular, high blood flow velocities in the middle cerebral artery are strongly associated with an increased risk of primary stroke [6].

Adams et al demonstrated that TCD could effectively be used to screen pediatric patients with SCA to identify those with an increased risk of primary stroke [6,7]. These landmark studies documented that the relative risk of stroke was much higher among children with an abnormal TCD, defined as the maximum time-averaged mean velocity (TAMV) ≥ 200 cm/sec. The stroke risk in children with SCA and abnormal TCD velocities, or those with conditional velocities (170-199 cm/sec), are 9% and 2% to 5% per year, respectively [6]. Age is also an important risk factor: among patients under 10 years of age, the overall risk of converting from a normal or conditional TCD velocity to an abnormal velocity is about 30% [8].

The predictive value of TCD velocities has led to its widespread use in screening children for primary stroke risk, and to guide therapy for stroke prevention. After TCD velocities cross the threshold of 200 cm/sec into the abnormal category, monthly transfusions are recommended to reduce stroke risk. [9,10]. However, this treatment modality is impractical in many developing countries where the blood supply may be inadequate, expensive, or unsafe. Challenges associated locally such as inadequate blood donations and high costs associated with chelation therapy to treat transfusion-acquired iron overload make chronic transfusion programs not feasible. Hence, an alternative therapy is needed for stroke prevention. Particularly for developing countries, the identification of an effective non-transfusion protocol for both primary and secondary stroke prevention in SCA is of critical importance.

Preliminary data have provided evidence that hydroxyurea provides neuroprotection and significantly reduces TCD velocities [11-14], and a small randomized prospective trial documented its efficacy in preventing conversion from the conditional to abnormal range [15]. Recent data also support the use of hydroxyurea as a substitute for chronic transfusion after at least one year of transfusion in children with SCA who have abnormal TCD without severe vasculopathy as defined by magnetic resonance angiography (MRA) in order to maintain TCD velocities and help prevent primary stroke [16]. However, a substantial knowledge gap exists regarding the role of hydroxyurea for newly diagnosed severe cerebrovascular disease, before the use of transfusions. No prospective trials have been conducted in this setting, although a retrospective review in Jamaica concluded that hydroxyurea could effectively prevent secondary stroke compared to observation alone [17]. Within the context of this background, and to address this critical knowledge gap the EXpanding Treatment for Existing Neurological Disease (EXTEND) trial was designed to investigate the effect of open label hydroxyurea on the maximum TAMV after 18 months of treatment compared to the pre-treatment value.

The authors believe that the publication of this protocol allows for an increased awareness of a successful working partnership between countries. The objective of this manuscript is to describe the protocol of this trial and provide a brief summary of results to date.

## Methods

### Study Design and Aims

The aim of EXTEND is to prospectively treat children with SCA who have either a conditional or abnormal TCD velocity with hydroxyurea in order to determine whether treatment is associated with altered velocity in the intracranial vessels, neurological outcomes, or quality of life (QOL) differences when compared to the pre-treatment state. It is an open-label trial designed to provide data on the safety and benefits of hydroxyurea in Jamaica. This is a Phase II clinical trial because the primary outcome, which is the effect of hydroxyurea on lowering elevated TCD velocities, is without a comparison arm. In this setting, it is neither practical nor ethical to include a control arm since regular blood transfusions for the treatment of cerebrovascular disease are not available in Jamaica. In addition, a prospective randomized controlled trial demonstrated that hydroxyurea could effectively lower conditional TCD velocities [15], and a retrospective study performed in Jamaica concluded that hydroxyurea could be beneficial for secondary stroke prevention [17]. The overall goal of the trial is to determine prospectively if hydroxyurea can serve as a protective treatment for neurovascular disease in children in a setting where the standard treatment of chronic blood transfusions is not practicable. EXTEND includes children with SCA and conditional TCD velocities, but also those with abnormal velocities and those who have already experienced a stroke, but who still have conditional or abnormal TCD velocities. Children who have experienced a stroke and maintain a conditional or abnormal TCD velocity will be eligible because a broad goal of EXTEND is to determine if hydroxyurea is a feasible treatment for neurovascular disease in children with SCA. Therefore, it is important to include even those on the most severe spectrum of disease, as there is no other treatment available. Only those with abnormal or conditional velocities are included to ensure the primary study endpoint can be evaluated in all participants.

The primary outcome measure is maximum TAMV obtained in the main intracranial arteries, typically the middle cerebral artery or distal internal carotid artery in both hemispheres. TCD velocities will be measured every 6 months, and the primary endpoint is the change in highest TAMV between the pre-hydroxyurea value and after 18 months of treatment. The Sparing Conversion to Abnormal TCD Elevation (SCATE) trial (NCT01531387) demonstrated that hydroxyurea could significantly reduce conditional TCD velocities within this timeframe [15].

Secondary endpoints of the EXTEND trial will include serial TCD velocity changes, incidence of neurological and non-neurological events, magnetic resonance imaging (MRI) and MRA changes of the brain, hydroxyurea-related toxicities and treatment responses, genetic and serological markers of disease severity and changes to QOL, neurodevelopment, body composition, resting metabolic rate, and lung function.

### Study Setting

Cincinnati Children’s Hospital Medical Center (CCHMC) serves as both the medical coordinating center (MCC) and the data coordinating center (DCC), while the Sickle Cell Unit (SCU) at UWI in Kingston, Jamaica is the clinical site. The SCU sees patients with SCA throughout their lifespan and has recently begun universal TCD screening to identify children at highest risk for stroke. The EXTEND protocol is approved by both the UWI Ethics Committee and the CCHMC institutional review board (IRB).

### Recruitment

**Figure 1:**
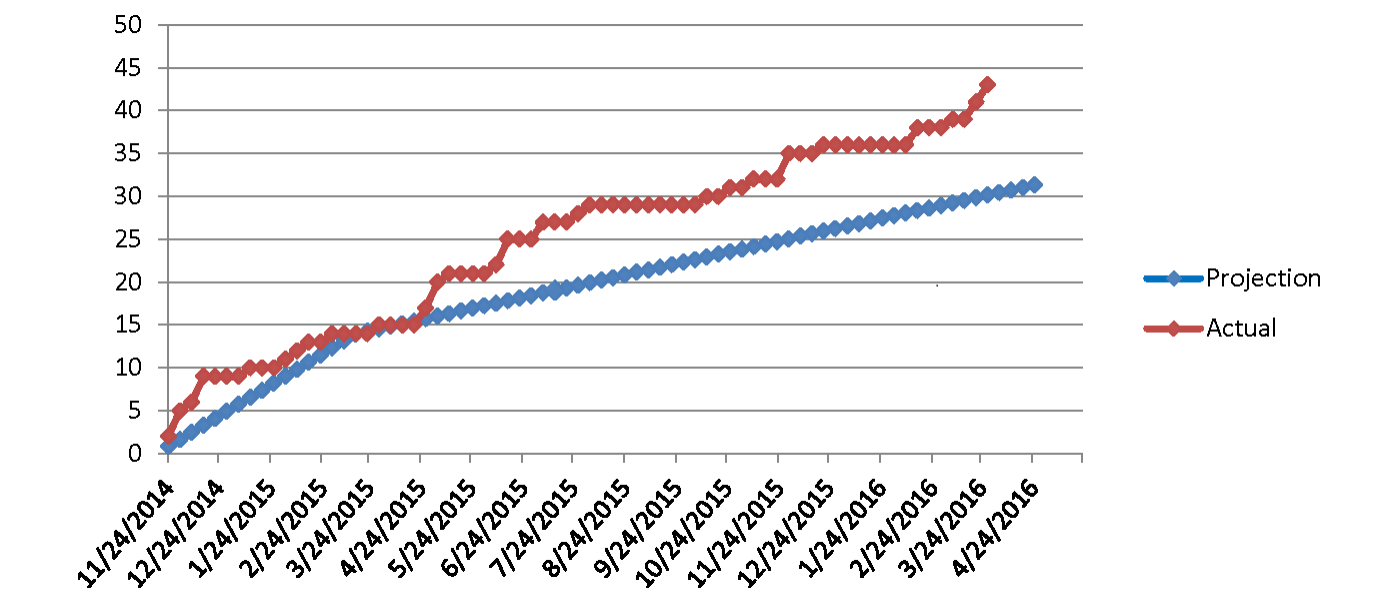
Actual and projected enrollment in the EXTEND study.

A maximum of 50 children will be recruited for the study. All children with SCA identified by routine TCD screening to have either conditional or abnormal TCD velocities are eligible for participation. The first enrollment into EXTEND was November 2014 with plans for completion during 2016; current enrollment is illustrated in Figure 1.

Specific inclusion criteria include: patients between ages 2 to 17 years with SCA (defined as genotypes HbSS, HbSβ^0^ thalassemia, HbSD, HbSO_Arab_), if their maximum TAMV is in the conditional (170-199 cm/sec) or abnormal (≥ 200 cm/sec) range by TCD ultrasonography within 6 months of enrollment. Key exclusion criteria include (1) participants who received an erythrocyte transfusion within the past 2 months; (2) the use of novel therapeutic agents within 3 months of enrollment; (3) known allergy to hydroxyurea therapy; and (4) positive human immunodeficiency virus (HIV) serology, malignancy, or other serious conditions. To protect against toxicity, patients with abnormal laboratory values on screening, defined by hemoglobin < 6 gm/dL, absolute reticulocyte count (ARC) < 100 x 10^9^/L, white cell count < 3.0 x 10^9^/L, or elevated serum creatinine will be temporarily excluded until the laboratory parameter improves.

### Study Treatment

Participants will receive open-label hydroxyurea, available as capsules (200 mg, 300 mg, 400 mg, or 500 mg), or as a liquid formulation (100 mg/mL) as described [18]. Hydroxyurea will be administered once daily by mouth. Participants will be monitored monthly with dose escalation to the maximum tolerated dose (MTD) and thereafter with quarterly clinical evaluations, laboratory tests, and semi-annual TCD examinations. Strategies for monitoring drug adherence include counting or measuring drug supply returned at each study visit, and participant and family report of adherence. During the trial, routine care for each participant will be continued in the regular sickle cell clinic.

Hydroxyurea dosing and escalation will occur as previously described [19]. Briefly, hydroxyurea treatment will commence at 20 mg/kg/day and be titrated to the MTD as defined by mild marrow suppression, even if the participant has clinical well-being at a lower hydroxyurea dose. The target absolute neutrophil count (ANC) on hydroxyurea therapy will be 1.0 to 3.0 x 10^9^/L, but the marrow suppression should also include reduction of the reticulocyte count [20]. Dose escalation will occur until the target suppression (ANC < 3.0 x 10^9^/L) is achieved. After reaching MTD, minor hydroxyurea dose adjustments can be made periodically, as necessary based on weight changes and blood counts, to maintain the optimal laboratory response and to prevent dose-related toxicity. Hydroxyurea will be temporarily discontinued for hematological toxicities with dose reductions for repeated or prolonged toxicities.

### Study Procedures

**Figure 2:**
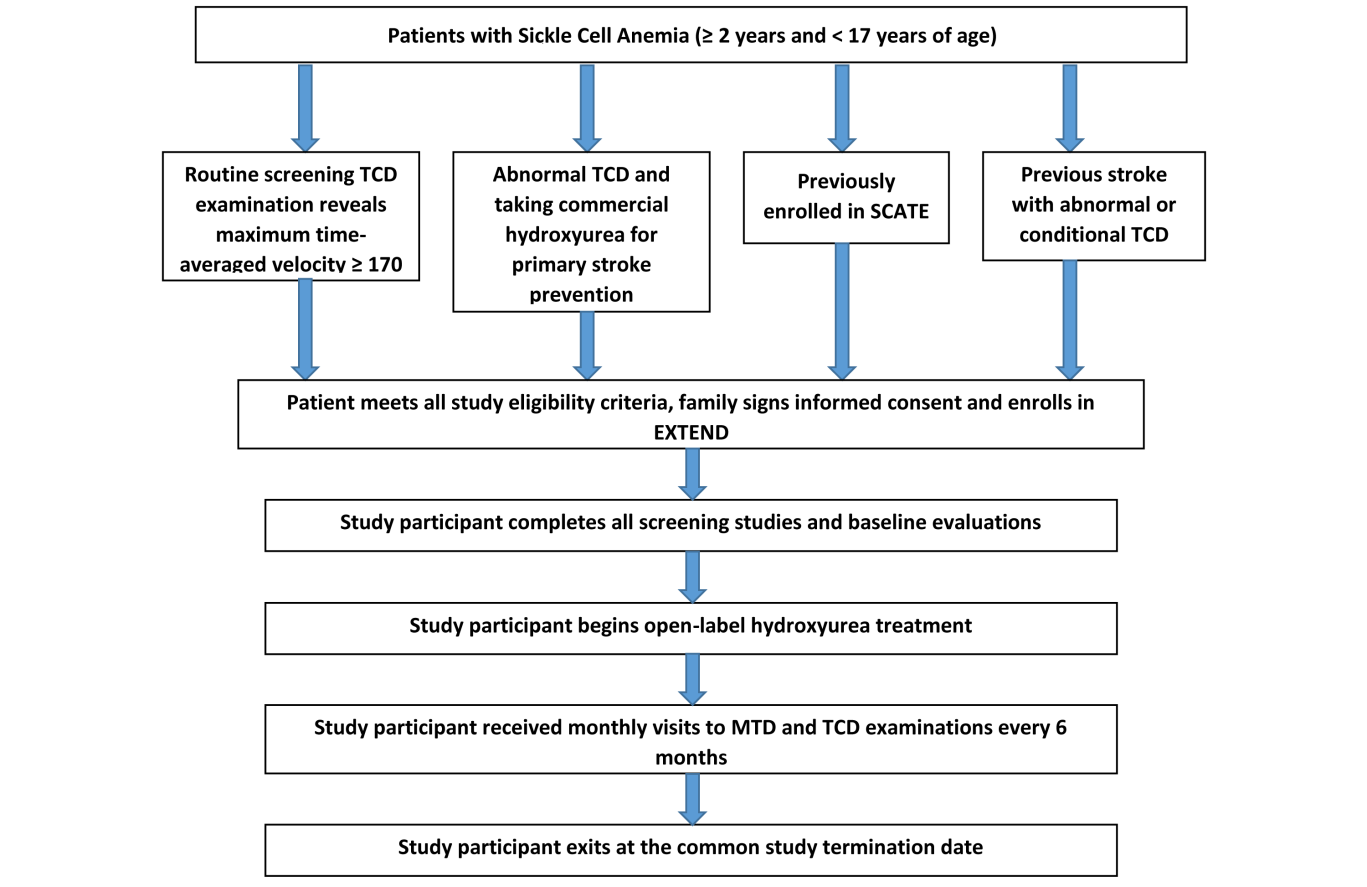
EXTEND study flow diagram.

The EXTEND recruitment and enrollment procedures, followed by the treatment and monitoring process is illustrated in Figure 2. Once the legal guardian provides informed consent and participants aged 12 to 17 years provide assent, study procedures will commence. Complete blood count (CBC), reticulocyte count, serum chemistries, and hemoglobin electrophoresis will be performed at the screening visit to evaluate eligibility. Additional assessments performed during the screening period include: TCD, comprehensive history and physical examination, vital signs and anthropometric measurements, neurological exam, and a pregnancy test if applicable.

Once the local site confirms eligibility of the participant, the following baseline visit assessments will be performed: (1) blood counts and chemistries as well as hemoglobin electrophoresis, (2) TCD, brain MRI/MRA, pulmonary function tests (PFTs), (3) QOL and neuropsychological assessments, and (4) metabolic studies of body composition, resting energy expenditure and protein metabolism. TCD examinations will be performed every 6 months and participants will remain on study treatment for at least 18 months, until a common study termination date approximately 3 years after the first enrollment.

### Study Evaluations

The TCD procedures in EXTEND will follow the Stroke Prevention in Children with Sickle Cell Anemia (STOP) study protocol [7,21]. All TCD examinations will be performed by a trained examiner using a non-duplex 2-MHz Doppler ultrasound machine (SONARA/tek, Natus Medical Inc., Middleton, Wisconsin). All TCD studies will be evaluated and scored locally, and the results entered into the EXTEND electronic database. There will be no central review of the TCD examinations, but 10% of scans will be reviewed every 6 months for quality control. The TCD coordinator from the MCC, who has been responsible for training TCD examiners and reading and reviewing research TCDs for over twenty years, will train and certify all EXTEND TCD examiners as well as perform quality control as described.

To more comprehensively evaluate neurovascular disease, brain MRI and MRA will be performed at study entry and after 18 months of study treatment for all children who receive study treatment. Brain MRI/MRA will also be performed to evaluate all acute neurological events experienced by EXTEND participants. Imaging sequences will include sagittal T1, axial T1, fluid attenuated inversion recovery (FLAIR), T2-weighted images coronal FLAIR, and diffusion weighted images on MRI, and MRA using three dimensional time of flight techniques, as described [22].

### Additional Study Measurements

The validated Pediatric Quality of Life Inventory (PedsQL) tool will measure QOL. Because the PedsQL 4.0 contains two components of child self-report and parent proxy-report, young patients will have the parent proxy-report data only. QOL will be measured at baseline, after 18 months of study treatment, and exit. Neurodevelopment will be measured at baseline and after 18 months of hydroxyurea treatment, using the standardized Wechsler Abbreviated Scale of Intelligence (WASI) neuropsychological assessment tool. PFTs including pulmonary function spirometry will be performed in age-appropriate children at baseline and after 18 months of study treatment. Lung volume will be measured using a helium gas dilution technique (Morgan TLC Test Mk 11, Morgan Scientific, Haverhill, MA) [23]. Resting energy expenditure will be measured using the indirect calorimetry method, and body composition by deuterium dilution. Protein metabolism will be measured using a prime continuous infusion of^13^C-phenylalanine in the fed state. All of these tests will be performed at study entry and again after 18 months of study treatment.

### Management of Data

Clinical data will be entered via the secure, Internet-based electronic data capture (EDC) system, OnCore, provided by the DCC. OnCore provides real-time data validation feedback, with an opportunity for real-time or delayed data corrections. The OnCore Protocol Management & Data Capture System provides tools to implement a wide variety of validation rules, such as edit checks, valid value and range checks as well as consistency checks. Validation rules will be applied whenever possible in the data entry and management process. Such reports will be generated at intervals that are useful to study leadership, the operations committee, the study monitor, and other applicable groups.

Queries are issued via the Data Monitor Console in the OnCore system, which is used as the validation and management workspace for online forms. As case report forms (CRFs) are completed, the monitor is able to validate the entered data and either issue a new query or lock the form. Issuing a query will send the form back to the person who entered the data with any questions associated with the query, whereas locking the form will preserve the data and prevent it from being altered once validated. This auditing will occur on an ongoing basis, and on-site monitoring visits will occur at least once per year.

### Statistical Analysis

For the primary endpoint, up to 50 children will be enrolled, depending on the screening efforts performed by the clinical site. No formal sample size calculations have been performed since EXTEND enrollment is based on available patients with conditional or abnormal TCD velocities. However, our projected sample size in EXTEND will allow us to detect an effect size of > 15 cm/sec for hydroxyurea treatment with an alpha of .05 and power of > 90%.

For secondary endpoints, the highest TAMV for each time period along with the baseline values will be analyzed using repeated measures analysis of variance (ANOVA) in order to model the potential efficacy of hydroxyurea in reducing elevated TCD velocities in a longitudinal manner. To compare the cumulative incidences of categorical changes of TCD velocity (eg, conditional to abnormal, or abnormal to normal), participants with drug adherence of ≥ 50%, determined by measuring monthly returned medication, will be analyzed to determine the percentage of children with either conversion or reversion of their TCD velocities. Baseline labs will be compared to the exit studies using Wilcoxon signed-rank tests, while clinical efficacy will be assessed by comparing the number of hospitalizations and transfusions during study treatment, compared to data prior to enrollment. The incidence of stroke, non-stroke neurological events, and non-neurological sickle-related events will also be compared.

Changes in QOL scores from baseline to study exit will be compared between treatment groups by the Friedman test. The sub-scores for each domain, including physical, emotional, social and school functioning will be analyzed individually and in aggregate. Finally, a preliminary analysis of the cost-effectiveness and clinical efficacy of hydroxyurea will be performed to evaluate whether treatment can serve as an effective front-line therapeutic alternative to blood transfusions for existing neurological disease among children with SCA living in developing countries where chronic transfusions are not always feasible or available.

## Results

A total of 43 participants have been enrolled. One participant withdrew from the study due to emigration outside of Jamaica. There have been no permanent screen failures. Of the participants, 37 have initiated study treatment; 36 participants are currently receiving study treatment, with plans to initiate all participants on treatment by July 2016. All participants will complete at least 18 months of study treatment before exiting the study at the common termination date. The early baseline results of this trial will be reported in early 2018. Final results will be reported in early 2019.

## Discussion

### Principal Findings

EXTEND is a continuation of the collaboration between CCH and UWI, officially established in 2012, but with beginnings prior because of a common interest in neurological complications of SCD by the lead investigators. The development of the partnership and SCATE protocol (NCT01531387) established a high quality TCD screening program in a large pediatric sickle cell population in Jamaica and provided the very first TCD findings in Jamaican children with SCD. The local health care team gained experience using hydroxyurea, which has improved the likelihood of future utilization in the general clinic population, particularly with regard to dosing and therapeutic monitoring. SCATE evaluated the effect of hydroxyurea compared to observation on conditional TCD velocities.

EXTEND is an innovative prospective trial that will evaluate the utility and efficacy of hydroxyurea in the setting of children with SCA and newly diagnosed cerebrovascular disease identified by conditional or abnormal TCD velocities. Building on a growing body of evidence, EXTEND aims to provide additional data to support the use of hydroxyurea as a treatment alternative to the current standard treatment of blood transfusions for cerebrovascular disease; this alternative is sorely needed in developing countries where access to safe blood transfusions is limited. If children with documented cerebrovascular disease are able to benefit from hydroxyurea without transfusion support, then TCD screening programs in developing countries can be developed without the requirement for safe and affordable blood. In addition, EXTEND will provide data on the benefits of hydroxyurea on other critical factors of health for children afflicted with SCD in Jamaica such as growth and nutrition, neurocognition, pulmonary function, and QOL. Importantly, any off-study treatment decisions should await completion of full data analyses.

### Conclusion

The EXTEND trial results will potentially have a large impact on the management of children with SCA on a worldwide scale. The study represents international collaborative research involving developing countries, which is a stated goal of the National Heart, Lung and Blood Institute and the subject of academic advocacy [24,25]. Beyond the specific study objectives, the development of such collaborations between resource-rich countries and the developing world has the potential to be of considerable benefit to the health and well-being of patients with SCA in those developing nations. In the case of EXTEND, it is therefore expected that a successful collaborative research study with Jamaica will advance research expertise and potentially improve clinical care for all children with SCA. Finally, EXTEND represents continued collaboration between an established sickle cell program at CCHMC and a distinguished international SCA program in Jamaica, which may lead the way for future collaborative studies.

## Abbreviations

ANC: absolute neutrophil count
ARC: absolute reticulocyte count
CCHMC: Cincinnati Children’s Hospital Medical Center
DCC: data coordinating center
EXTEND: EXpanding Treatment for Existing Neurological Disease
FLAIR: fluid attenuated inversion recovery
HbSS: homozygous sickle cell anemia
MCC: medical coordinating center
MCV: mean cell volume
MRA: magnetic resonance angiography
MRI: magnetic resonance imaging
MTD: maximum tolerated dose
PedsQL: Pediatric Quality of Life Inventory
PFT: pulmonary function test
QOL: quality of life
SCA: sickle cell anemia
SCD: sickle cell disease
SCU: Sickle Cell Unit
TAMV: time-averaged mean velocity
TCD: transcranial Doppler
UWI: University of West Indies
